# Analysis of esterase enzyme activity in adults of the major malaria vector *Anopheles funestus*

**DOI:** 10.1186/s13071-016-1379-7

**Published:** 2016-02-27

**Authors:** Yael Leah Dahan-Moss, Lizette Leonie Koekemoer

**Affiliations:** Wits Research Institute for Malaria, School of Pathology, Faculty of Health Sciences, University of the Witwatersrand, Johannesburg, South Africa; Centre for Opportunistic, Tropical and Hospital Infections, National Institute for Communicable Diseases, a Division of the National Health Laboratory Service, Sandringham, Johannesburg, South Africa

**Keywords:** *Anopheles funestus*, Isoenzyme electrophoresis, Esterase classification

## Abstract

**Background:**

*Anopheles funestus* is a major vector of malaria in sub-Saharan Africa. In order to apply effective control measures against this vector, it is necessary to understand the underlying physiological factors that play a critical role in its development, reproduction, fertility and susceptibility to insecticides. One enzyme family involved in the above mentioned biological pathways is the esterases. The aim of this study was to analyse esterase activity levels at different ages during the life-span of adult *Anopheles funestus* Giles in order to better understand the complex biological processes in this species.

**Methods:**

Isoenzyme electrophoresis (IEE) was used to examine the esterase activity in laboratory colonised *An. funestus* adults aged between 2 h (h) and 30 days post eclosion as well as in wild *An. funestus* adults aged between 2 h and 15 days post eclosion. Esterase activity was quantified by densitometry analysis of the IEE gels. Esterases were classified according to their activity inhibition by organic phosphates, eserine sulphate and sulphydryl reagents.

**Results:**

Nine esterases IEE profiles were common to both the laboratory colonised and wild *An. funestus* adults. These esterases were further divided into acetylesterases, arylesterases, carboxylesterases and acetylcholinesterase. The activity level of certain specific esterases was primarily influenced by age and/or gender.

**Conclusions:**

The information from this study contributes towards the general understanding of esterase enzyme activity variation in adults of a major malaria vector *An. funestus*. This variation likely carries physiological and adaptive significance and may influence specific characteristics, such as reproductive fitness and insecticide resistance that are epidemiologically important.

## Background

Malaria is a devastating vector-borne disease, which in 2015 resulted in an estimated 438,000 deaths worldwide, the majority of which occurred in sub-Saharan Africa [1]. *Anopheles funestus* is a major malaria vector in sub-Saharan Africa and is capable of causing severe epidemics such as that experienced in South Africa from 1996 to 2000 [2, 3]. During the past decade, several studies have highlighted the increasing prevalence of resistance to insecticides in *An. funestus* populations [4–6] and the resultant threat posed to effective vector control. However, there have been few studies covering other important aspects of *An. funestus* s.s. biology.

The application of effective vector control measures against insect disease vectors is best achieved using an understanding of the reproductive, behavioural and ecological characteristics of each species as well as the underlying physiological processes that govern these characteristics. Enzyme expression, including variation in expression by life stage and age, is important in the mediation of these characteristics. The esterase family consists of numerous enzymes all of which catalyse the hydrolysis of esters. In insects, esterases are linked with critical physiological roles such as behaviour [7], development [8–10], insecticide resistance [11–13], and reproduction [14].

Esterases in arthropods including *Anopheles* can be classified according to alpha or beta esterases depending on their ability to hydrolyze the substrates alpha- and beta-naphthyl acetate respectively [11, 15, 16]. In addition, esterases can be characterized according to inhibition criteria [13, 15, 17, 18]. This is based on the use of organophosphates to inhibit carboxylesterase activity, organophosphates and eserine sulphate to inhibit cholinesterase activity and sulphydryl reagents to inhibit arylesterase activity. The acetylesterases are not inhibited by the above-mentioned compounds [13].

Esterase related studies in *An. funestus* adults have been restricted to their role in insecticide resistance and establishing the presence of a sex-limited esterase in the male accessory glands [19–21]. Increased esterase activity was recorded in pyrethroid and carbamate resistant *An. funestus* adults from the Tihuquine region in Mozambique [19], as well as in specimens from the Chikwawa region in Malawi which were resistant to permethrin, deltamethrin, bendiocarb and DDT [20]. In a different study relating to *An. funestus* esterases, Green demonstrated that there is a specific esterase that is mainly concentrated in male *An. funestus* accessory glands [21]. Although these studies added insights to the *An. funestus* physiology from an esterase perspective, it is necessary to further examine the esterase activity profile in *An. funestus* adults to better understand biological processes in this important vector species. The aim of this study was to characterise the different esterases in the *An. funestus* adults to provide new knowledge on this species biology and possibly its control.

## Methods

### Laboratory colonised and wild An. funestus samples

Samples of both laboratory-reared and wild caught *An. funestus* s.s. females and males were used in this investigation. All were housed in the Botha de Meillon Insectary of the National Institute for Communicable Diseases (NICD), Johannesburg, and maintained under standard insectary conditions of 25 ± 1 °C and 80 % relative humidity with a 12:12 h light/dark cycle and 45 min dusk/dawn simulation. The FUMOZ laboratory colony was established in 2000 using wild-caught *An. funestus* material from southern Mozambique [22]. The wild *An. funestus* material (FUZIM) was obtained from adult *An. funestus* females collected from houses in Honde Valley, Zimbabwe (18°25’19.0”S, 32°58’35.8”E), in March 2014. They were transported back to the NICD and induced to lay eggs. Eggs were reared through to F_1_ adults, which were used for this study.

Emerging FUMOZ and F_1_ FUZIM *An. funestus* females and males were collected at 30 min intervals and placed in 2 L cages supplemented with a 10 % sucrose solution. FUMOZ adults (aged between 2 h to 30 days) and FUZIM adults (aged between 2 h and 15 days) were removed from the cages at specific times and stored at−70 °C until they were prepared for electrophoresis analysis. Due to the limited number of F_1_ FUZIM samples, only adults that were aged between 2 h to 15 days old were used.

### Isoenzyme electrophoresis

For each time point, two *An. funestus* adults of each sex and from the FUMOZ and F_1_ FUZIM populations were homogenized in 80 μl of grinding buffer (10 % sucrose, 33 % Tris-Boric acid-EDTA (TBE) buffer, pH 8.9) [23]. The homogenates were centrifuged at 13 000 rpm for 5 min and 8 μl of the supernatant was loaded onto a continuous non-denaturing native 7.5 % polyacrylamide gel. The homogenates were electrophoresed for 3 h at a constant ampage of 40 mA with continuous cooling at 4 °C. Subsequently, the gels were incubated in a soaking solution (0.1 M sodium phosphate buffer, pH 6.4, which contained 8 % of alpha or beta naphthyl solution (2 % of alpha or beta naphthyl acetate in 50 % acetone)) at 37 °C for 30 min. The gels were then stained in a soaking solution containing 0.1 % Fast blue R/R at 37 °C for 2 h. ImageJ software [24] was used to perform densitometry analysis of the IEE gels, which provided an estimation of the esterase activity.

### Characterization of esterases by inhibitory assays

The esterases were characterised according to their inhibition criteria [13]. Isoenzyme electrophoresis was performed as described above with the exception that individual gels were placed in a soaking solution containing one of the following inhibitors: p-hydroxymercuribenzoic acid sodium salt (pHMB, 1.5 mM); malathion (1.5 mM); eserine (1.5 mM) and phenylmethylsulfonyl fluoride (pMSF, 1.5 mM). Prior to adding pHMB and pMSF to the soaking solutions, pHMB was dissolved in 100 μl of NaOH, pH 9 and the PMSF was dissolved in 500 μl of absolute ethanol (Purity (GC) ≥ 99.5 %).

## Results

### Common esterases in the laboratory colonised and wild An. funestus adults

**Fig. 1:**
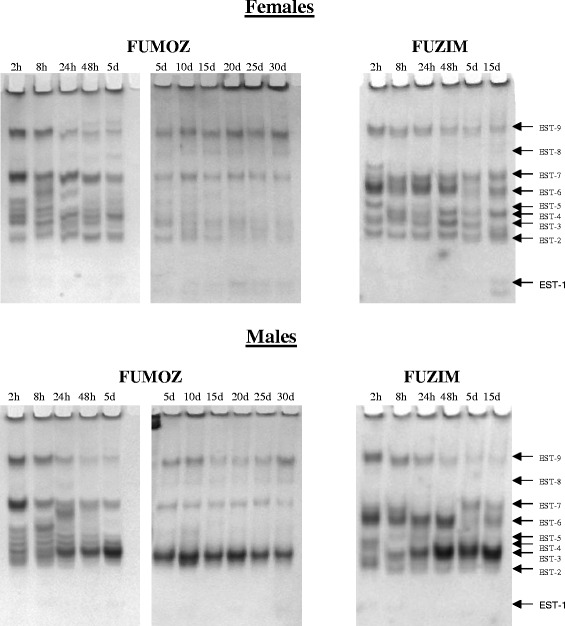
IEE gels depicting the activity of esterases 1 to 9 in the adult *An. funestus*. The FUMOZ females and males were aged 2 h to 30 days post eclosion and the F_1_ FUZIM *An. funestus* females and males were aged 2 h to 15 days post eclosion. (*h* = hours old; *d* = days old)

The electrophoretic pattern of the esterase activity in the *An. funestus* females and males from the FUMOZ colony that were aged between 2 h and 30 days post eclosion and the F_1_ FUZIM samples aged between 2 h and 15 days post eclosion is shown in Fig. 1. There were 9 electrophoretic bands of esterase isoenzymes on the IEE gel that were common to the FUMOZ and FUZIM samples. The esterase bands on the polyacrylamide gel were labelled EST-1 to -9 starting from the anode end of the gel (Fig. 1).

### Characterization of esterases 1 to 9

Esterases 1 to 9 were categorized as alpha and/or beta esterases according to their ability to hydrolyse either alpha- or beta-naphthyl acetate, which is indicated by the appearance of black or red bands on the IEE gels respectively. Esterases 1 to 9 all preferentially hydrolyzed alpha-naphthyl acetate as their activity was seen as a black band on the gel that had both alpha- and beta-naphthyl acetate as substrates. Esterase 2, 3, 5, 6, 7 and 9 were also able to hydrolyse beta-naphthyl acetate on a gel that had only beta-naphthyl acetate as a substrate.

The esterases were further characterized according to inhibition criterion. Esterase 1 and 8 were not inhibited by eserine sulphate, malathion or pHMB (Table 1). Esterase 2 and 4 were strongly inhibited by pHMB. Esterase 3 activity was strongly inhibited by PMSF and slightly inhibited by pHMB. Esterases 5, 6 and 7 were inhibited by both malathion and PMSF and EST-9 was strongly inhibited by eserine sulphate and pHMB (Table 1).

**Table 1:** The inhibition of *An. funestus* esterases 1 to 9 with specific substrates

Esterase	Eserine	Malathion	pHMB	PMSF
EST-1	-	-	-	-
EST-2	-	-	+ + +	-
EST-3	-	-	+	+ + +
EST-4	-	-	+ + +	-
EST-5	-	+ + +	-	+ + +
EST-6	-	+ + +	-	+ + +
EST-7	-	+ + +	-	+ + +
EST-8	-	-	-	-
EST-9	+ + +	-	+ + +	-

- = no inhibition of esterase activity, + = slight inhibition of esterase activity and +++ = strong inhibition of esterase activity. *PHMB* p-hydroxymercuribenzoic acid sodium salt; *PMSF* phenylmethylsulfonyl fluoride

### Analysis of esterase activity in adult *An. funestus* females and males

**Fig. 2:**
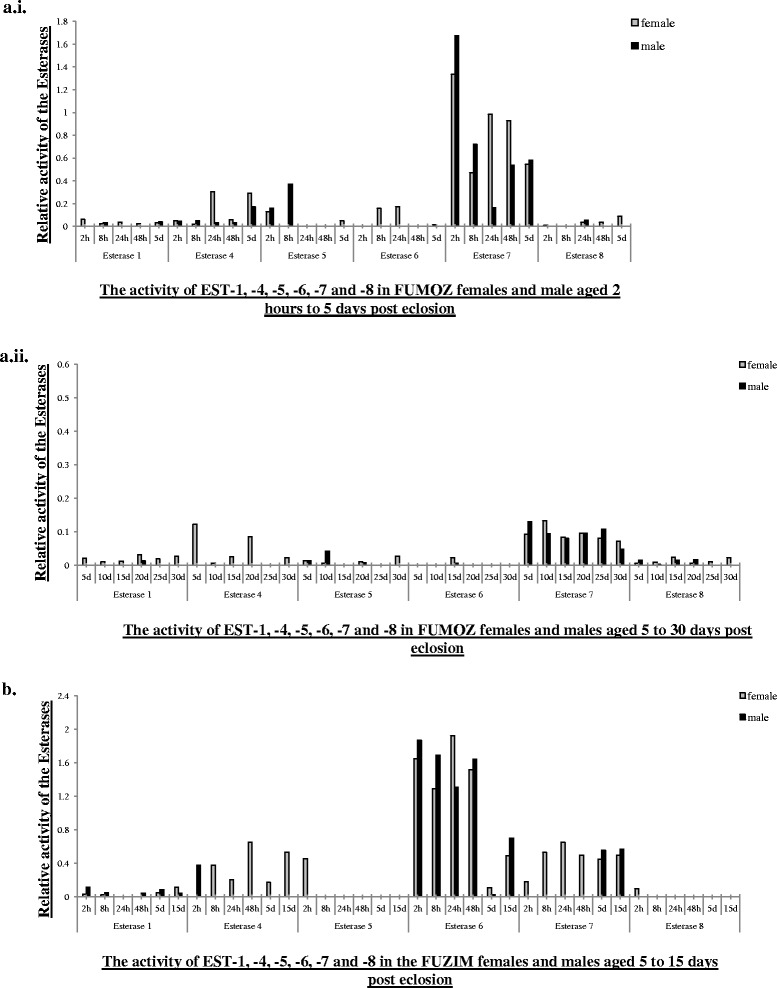
The activity of EST-1,-4,-5,-6,-7 and-8 in the adult *An. funestus*. **a.i**) FUMOZ female and males aged 2 h to 5 days post eclosion; **a.ii**) FUMOZ female and males 5 to 30 days post eclosion; **b**) FUZIM females and males aged 2 h to 15 days post eclosion. (*h* = hours old; *d* = days old). The esterase activity was standardized according to the EST-9 activity observed in newly emerged (2 h) females in of the FUMOZ and FUZIM males, which was set at 1 fold

**Fig. 3:**
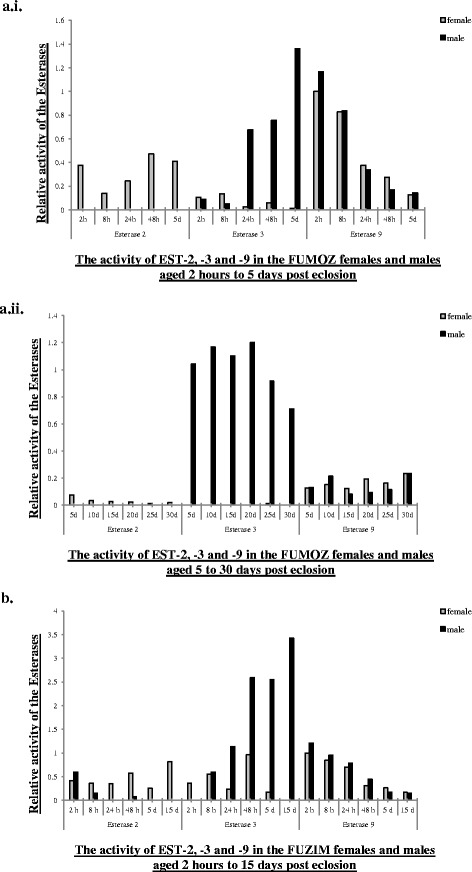
The activity of EST-2,-3 and-9 in the adult *An. funestus*. **a.i**) FUMOZ female and males aged 2 h to 5 days post eclosion; **a.ii**) FUMOZ female and males 5 to 30 days post eclosion; **b**) FUZIM females and males aged 2 h to 15 days post eclosion. (*h* = hours old; *d* = days old). The esterase activity was standardized according to the EST-9 activity observed in newly emerged (2 h) females in FUMOZ and FUZIM males, which was set at 1 fold

Densitometry of the IEE gels was used for the semi-quantitative analysis of the activity of the different esterases in all samples. The activity of EST-1, EST-4, EST-5, EST-6, EST-7 and EST-8 fluctuated at the different ages and sexes (Figs. 1 and 2). In contrast, EST-2, EST-3 and EST-9 showed a specific pattern of esterolytic activity that can be linked to sex and/or age (Fig. 3).

EST-2 activity was evident in each of the laboratory-reared FUMOZ females that were aged 2 h (h) to 30 days and the FUZIM females aged 2 h to 15 days (Figs. 1 and 3). In contrast, the activity of this esterase was not evident in the FUMOZ males aged 2 h to 30 days. EST-2 activity was only detectable in FUZIM males that were aged 2 h, 8 h and 48 h post eclosion. EST-2 activity was 2.4 and 7.3 fold higher in the FUZIM females aged 8 h and 48 h than males of the same age (Figs. 1 and 3).

EST-3 activity was higher in the FUMOZ males aged 24 h to 30 days post eclosion and FUZIM males aged 24 h to 15 days post eclosion versus corresponding females from the same colonies and of the same ages (Figs. 1 and 3). The activity of EST-3 ranged between 12.9 and 114.7 fold higher in FUMOZ males aged 24 h to 30 days in comparison to EST-3 activity in the females of the same ages. Additionally, EST-3 activity was barely detectable in the FUMOZ females aged 5 days to 30 days. The fold difference of EST-3 activity in FUZIM was between 2.7 and 14.7 higher in males aged 24 h to 15 days when compared to the females of the same ages. Furthermore, EST-3 activity was not detectable in FUZIM females aged 15 days post eclosion (Figs. 1 and 3). EST-3 activity in adult females from FUMOZ and FUZIM was variable (Figs. 1 and 3). In contrast, it was notable that EST-3 activity increased with age in FUMOZ (aged 2 h to 5 days) and FUZIM males (aged 2 h to 48 h). The EST-3 activity was 15.1 fold higher in FUMOZ males that were 15 days old than in newly emerged males (2 h) (Figs. 1 and 3). After 5 days the activity of EST-3 in the males was variable with the highest fold difference in activity being 1.7 (Figs. 1 and 3). Similarly, in FUZIM males EST-3 activity was not detected in newly emerged (2 h) males, while the increase in EST-3 activity in 48 h males versus 8 h males was 4.4 fold. After 48 h, the highest fold difference in EST-3 activity in the FUZIM males was 1.3.

Esterase 9 activity was detected in females and males from FUMOZ and FUZIM at every age examined between 2 h and 30 days (Figs. 1 and 3). The esterolytic activity of EST-9 in laboratory colonised and wild *An. funestus* was at its maximum during the period that directly follows emergence (i.e., at 2 h) (Figs. 1 and 3). The fold decrease of EST-9 activity between 2 h and 8 h old FUMOZ females and males was 1.2 and 1.4 fold respectively. Furthermore, the EST-9 activity decreased in the FUMOZ *An. funestus* adults that were aged 2 h versus 24 h to 30 days by a range of 2.7 to 8.1 fold in the females and between 3.5 and 14.4 fold in the males. In the FUZIM adults, the EST-9 activity reduced by 1.2 and 1.3 fold in the females and males aged 2 h versus 8 h. The fold decrease in EST-9 activity of FUZIM females and males aged 2 h versus 24 h to 15 days was between 1.4 and 5.9 fold in FUZIM females and between 1.5 and 7.8 fold in FUZIM males (Figs. 1 and 3).

## Discussion

Nine specific esterases were evident in the *An. funestus* adults sampled regardless of whether their genotypes originated from southern Mozambique (FUMOZ) or Honde Valley, Zimbabwe (FUZIM). Of these esterases EST-1, -4 and -8 were classified as alpha esterases and EST-2, -3, -5,-6, -7 and -9 as alpha-beta esterases based on their abilities to hydrolyse alpha- or beta-naphthyl acetate. Esterases 1 to 9 were further characterized according to inhibition criteria [13]. Esterases 1 and 8 were acetylesterases, as their activities were not inhibited by eserine sulphate, malathion or pHMB. Esterase 2 and 4 were arylesterases as they were only inhibited by pHMB. Esterases -3, -5, -6 and -7, which likely have serine residues in their catalytic active sites, were inhibited by PMSF. Esterases -5, -6 and -7 were also inhibited by malathion and were therefore classified as carboxylesterases with a serine residue in the active site which is common in the carboxylesterases of insects. Since EST-3 activity was strongly inhibited only by PMSF and very slightly by pHMB but not by malathion or eserine sulphate, this esterase was considered to be an acetylesterase. Esterase 9 was classified as an acetylcholinesterase due to the strong inhibition of its activity by eserine sulphate. In several insect species, including *An. gambiae* Giles, there is an acetylcholinesterase with a cysteine residue near or at the rim of its active site, which is inhibited by sulphydryl reagents [25–27]. It is very likely that this is also the case for EST-9 in *An. funestus*.

The pattern of esterolytic activity of the esterases in *An. funestus* as well as information of these esterases in other members of the class Insecta may provide a clue as to their function. The variability observed in EST-1, EST-4, -5, -6, -7 and -8 could be due to the influence of external or environmental factors such as fluctuating temperature and humidity or could reflect the influence of the available food source on the expression and activity of these esterases. This suggests that during the lifespan of adult *An. funestus* these esterases play an important role in environmental adaptation.

The *An. funestus* acetylcholinesterase EST-9 was present at all the ages in males and females from both the colonies, indicating that the activity of this esterase is essential during the adult life of *An. funestus*. In insects, acetycholinesterase activity is important for neurotransmission [28], coordination, locomotor activity and development [29, 30], longevity, female reproduction and growth of offspring [30], as well as for developing insecticide resistance [31]. It is very likely that EST-9 activity in *An. funestus* has similar roles to those in other insects, which would be necessary to maintain the normal physiological functions in the adult stage.

The *An. funestus* acetylesterases EST-1, -8 and -3 showed variable esterolytic activity patterns at different ages and sexes. Therefore it is probable that these esterases, which are responsible for the hydrolysis of acetic esters, each play different roles in *An. funestus*. Mateus et al. proposed that the acetylesterase (EST-4) in *Drosophila* species (*D. mulleri*, *D. aldrichi*, *D. wheeleri*, *D. mojavensis, D. arizonae* and *D. navojoa*) may act as juvenile hormone (JH) esterases (JHE) [32]. Juvenile hormone plays a significant role in male sexual development in insects and is important for male reproductive fitness [33], sexual signalling [34], male mating behaviour and protein synthesis in the male accessory glands [35]. Since EST-3 is an acetylesterase and has a male specific esterolytic activity pattern in *An. funestus* ([21], herein), it is possible that EST-3 has a JHE or JHE-like activity, which would regulate the JH expression level in *An. funestus* males.

The arylesterases, EST-2 and EST-4 also showed differential esterolytic activity patterns. *Drosophila melanogaster* transfected with the human paraoxonase (PON) 1 gene led to an increase of arylesterase activity in the PON1 transgenic flies, which also resulted in the protection of these flies from *Pseudomonas aeruginosa* induced lethality [36]. *Pseudomonas* species are present in the midgut of many mosquitoes, including *Anopheles* [37]. Therefore, it is possible that the arylesterases in *An. funestus* may reduce the virulence of *Pseudomonas* species. Esterase-A (=arylesterase) was specifically found in the female salivary glands of *An. stephensi* Liston [38], supporting data presented here where EST-2 activity was predominantly higher in *An. funestus* females than males. If EST-2 is present only in the salivary glands of *An. funestus* females, it may further indicate an important role of this esterase during blood feeding or transmission of *Plasmodium* sporozoites. This needs to be further investigated.

The activities of carboxylesterases EST-5, −6 and EST-7 fluctuated in FUMOZ adults that were between 2 h and 30 days old and in FUZIM adults that were between 2 h and 15 days old. Carboxylesterases in other insects including *Anopheles* species play important roles in olfaction, behaviour, reproduction [7], insecticide resistance [39] pheromone degradation [40]. It is highly likely that these enzymes are involved in at least one of these functions. This would result in specific physiological changes that are necessary for important events during the lifespan of adult *An. funestus*.

## Conclusions

We confirmed the presence of nine esterases characteristic to *An. funestus* adults as detected by IEE in a long-established laboratory colony and a recent colony representing a wild *An. funestus* population. This is the first paper evaluating esterase activity over adult life span as well as the characterisation of esterase based on the inhibition criterion. Esterase-1 (acetylesterase),-4 (arylesterase),-5 (carboxylesterase),-6 (carboxylesterase),-7 (carboxylesterase) and-8 (acetylesterase) activities varied at different ages of adult *An. funestus* males and females and between colonies. Esterase-2 (arylesterase) and-3 (acetylesterase) activities at different ages were most pronounced in females and males respectively. Esterase-9 (acetylcholinesterase) activity was evident at the different ages in males and females.

The information from this study provides a stepping stone towards understanding enzyme-mediated physiological processes in adult *An. funestus* based on the esterase enzymes that are involved. Future studies regarding the activities of the esterases in *An. funestus* adults can be used to determine the structure and function of these esterases. Ultimately, these esterases could serve as valuable tools in understanding the biology of this species, which is essential for effective control of this major African malaria vector.

## Abbreviations

DDT: Dichlorodiphenyltrichloroethane
EST: Esterase
FUMOZ: *An. funestus* colony originating from Mozambique
FUZIM: Wild-caught *An. funestus* from Zimbabwe
IEE: Isoenzyme electrophoresis
JH: Juvenile hormone
JHE: Juvenile hormone esterase
NICD: National Institute of Communicable Diseases
pHMB: p-hydroxymercuribenzoic acid sodium salt
pMSF: Phenylmethylsulfonyl fluoride
PON: Paraoxonase
TBE: Tris-Boric acid-EDTA
